# Quantitative susceptibility mapping shows alterations of brain iron content in children with autism spectrum disorder: a whole-brain analysis

**DOI:** 10.1186/s12888-025-07235-y

**Published:** 2025-08-27

**Authors:** Xiaowen Xu, Yang Li, Haifei Lan, Ning Ding, Weikai Li, Guifen Zheng, Xiufeng Song

**Affiliations:** 1 Department of Radiology, Qingdao University Affiliated Women and Children’s Hospital, 6 Tongfu Road, Qingdao, 266034 Shandong China; 2 Department of Radiology, Peking University People’s Hospital Qingdao Hospital, 7 Jinsheng 1 st Road, Qingdao, 266000 Shandong China; 3 Department of Psychology, Qingdao University Affiliated Women and Children’s Hospital, 6 Tongfu Road, Qingdao, 266034 Shandong China

**Keywords:** Magnetic resonance imaging, Quantitative susceptibility mapping, Brain iron, Autism spectrum disorder

## Abstract

**Background:**

Iron deficiency in subcortical structures has been reported in previous studies using manually drawn regions of interest (ROIs). However, no whole-brain iron content studies in individuals with autism spectrum disorder (ASD) have been published. This study aimed to explore whole-brain iron content in ASD children using quantitative susceptibility mapping (QSM) and to examine relationships between clinical features of ASD and regional susceptibility values.

**Methods:**

A total of 30 ASD children and 28 typically developing (TD) individuals who were matched for age and sex were prospectively recruited. Brain MRI scans were performed on each participant. Each brain region’s susceptibility value was compared between groups, and correlations with clinical manifestations were examined.

**Results:**

The ASD patients showed significantly higher susceptibility values than TD children in the bilateral middle temporal gyrus, left inferior temporal gyrus, left inferior parietal gyrus, right lateral occipital gyrus, right insula, and bilateral rostral anterior cingulate gyrus. Conversely, significantly lower susceptibility was observed in the right cerebral white matter of ASD children. According to correlation analysis, susceptibility values in the left middle temporal gyrus, left inferior parietal gyrus, and right lateral occipital gyrus were negatively correlated with the Gesell Developmental Schedules (GDS) gross motor scores in the ASD group.

**Conclusions:**

ASD children had aberrant susceptibility values in cortical areas, and these abnormalities might be associated with their clinical features, which may provide new insights into understanding the pathophysiology of ASD.

**Supplementary Information:**

The online version contains supplementary material available at 10.1186/s12888-025-07235-y.

## Background

Autism spectrum disorder (ASD) is a lifelong neurodevelopmental disorder present at birth, though typically detectable around age two using current diagnostic methods. It is characterized by core features including impaired social communication and interaction, along with restricted, repetitive patterns of behavior, interests, or activities. These features can impact individuals’ lives and present challenges for families and society [1, 2]. ASD prevalence is increasing annually, with a global median prevalence of 1% in children [3]. According to the 2023 Centers for Disease Control and Prevention surveillance data, the prevalence among children aged 4 and 8 years was 2.15% and 2.76%, respectively [4, 5], while in China, the ASD prevalence in children aged 6–12 years was 0.7% [6].

Despite extensive research, the underlying mechanisms of ASD remain incompletely understood [7]. A recent study suggested that specific genetic differences in iron metabolism may play a significant role in ASD pathogenesis and could serve as potential early biomarkers for diagnosis [8]. Iron, an essential mineral, is crucial for brain development in children. Iron-containing enzymes and proteins are essential for neurotransmitter metabolism, neuronal myelination, and dendrite and synapse development [9, 10]. Alterations in iron metabolism may disrupt systemic homeostasis, as both iron overload and deficiency can cause functional impairment. Multiple studies indicate that dysregulated iron metabolism is associated with neurodevelopmental and psychiatric disorders; abnormal iron content has been detected in brains of individuals with depression, schizophrenia, Tourette syndrome, and attention-deficit/hyperactivity disorder [9, 11–14]. Therefore, non-invasive quantification of regional brain iron levels and investigation of their correlation with ASD clinical features are important for understanding ASD pathophysiology.

Quantitative susceptibility mapping (QSM), a novel MRI technique, calculates tissue magnetic susceptibility using phase information from multi-echo gradient echo sequences [15]. The susceptibility values derived from QSM demonstrate high correlation with Perls’ iron staining and direct iron measurements using inductively coupled plasma mass spectrometry or X-ray fluorescence imaging [16–19]. In cerebral gray matter, QSM quantifies susceptibility changes that primarily reflect iron content variations, enabling precise whole-brain iron mapping [20, 21]. Although some studies using manually drawn regions of interest (ROIs) have revealed iron alterations in subcortical structures, no published research has examined whole-brain iron deposition changes in ASD individuals [22, 23]. Thus, we aim to quantify whole-brain iron content in ASD children using QSM and assess relationships between ASD clinical features and brain areas showing altered iron deposition.

## Methods

### Participant information

Our institution’s ethics committee has approved the study (lot number: QDU-HEC-2022253). Every subject’s guardian signed an informed consent form and provided consent to the study.

Between January 2023 and April 2024, a group comprising 30 ASD children who received their initial diagnosis in the psychology department of Qingdao University Affiliated Women and Children’s Hospital was prospectively enrolled (ASD group). Concurrently, 28 typically developing (TD) children, matched for age and sex, who underwent a physical examination at the same hospital, were prospectively recruited (TD group). All the subjects were scanned using the enhanced susceptibility-weighted angiography (ESWAN) sequence and the 3D-T1WI sequence.

Inclusion criteria for ASD individuals were: being between the ages of 2 and 6; being right-handed; and fulfilling the diagnostic criteria for ASD in the American Diagnostic and Statistical Manual of Mental Disorders (Fifth Edition) (DSM-5). The following criteria were used to exclude ASD participants: failure to cooperate with MRI testing; any history of cerebral hemorrhage, brain infection, or brain trauma; and any history of neurological illnesses, genetic diseases, other mental disorders, or neuropsychiatric medication.

TD children were included if the participant met the following criteria: between the ages of 2 and 6; normal brain development; right-handed; and no discernible abnormalities on conventional brain MRI. The TD children were excluded based on the following criteria: not cooperating with MRI examination; any history of cerebral hemorrhage, brain infection, or brain trauma; any history of neurological illnesses, genetic diseases, other mental disorders, or neuropsychiatric medication; and any first-degree relative with autism spectrum disorder or other neurodevelopmental disorders.

### Clinical assessment

The DSM-5, which includes five items (A, B, C, D, and E), was used to diagnose ASD children. Item A, with three criteria, assesses deficiencies in communication and social interaction; item B, with four criteria, assesses restricted, repetitive patterns of behavior, interests, or activities; item C highlights the early onset of these symptoms; item D emphasizes their significant impact on daily functioning; and item E specifies that the symptoms are not better explained by intellectual disability or global developmental delay. The diagnosis of ASD was confirmed when the criteria for item A (requiring all three symptoms), B (requiring at least two of four symptoms), C, D, and E were met, as determined by simultaneous diagnostic agreement between two professional psychologists.

The Chinese version of the Gesell Developmental Schedules (GDS) was administered by a professional psychologist to evaluate the developmental quotient (DQ) in ASD children across five domains: adaptive behavior, gross motor, fine motor, language, and personal-social behavior. Lower DQ scores indicate poorer levels of brain development in each domain. Based on DQ scores, ASD children were classified into the following categories: very severe developmental delay (DQ < 25); severe developmental delay (25 ≤ DQ ≤ 39); moderate developmental delay (40 ≤ DQ ≤ 54); mild developmental delay (55 ≤ DQ ≤ 75); and marginal state (76 ≤ DQ ≤ 85).

### Image acquisition and processing

This study utilized a 3.0-T MRI system (SIGNA Architect; GE Healthcare) equipped with a 19-channel combined head and neck coil for image acquisition. All participants received 10% chloral hydrate for sedation (0.5 ml/kg) 30 min prior to examination to minimize motion artifacts and guarantee image quality. After the subjects fell asleep, they were placed on the MRI scanning bed with warmth and hearing protection. All children underwent conventional head MRI, 3D-T1WI, and ESWAN sequences. The following scan parameters were used to acquire the 3D-T1WI images: repetition time (TR) = 7.9 ms, echo time (TE) = 3.1 ms, flip angle (FA) = 12°, field of view (FOV) = 256 mm × 256 mm, and slice thickness = 1.0 mm. The ESWAN images were acquired using a three-dimensional gradient-echo sequence: TR = 24.8 ms, TE = 3.8 ms, FA = 20°, FOV = 256 mm × 256 mm, and slice thickness = 1.0 mm. Additionally, transverse axial DWI, T2 FLAIR, and T2WI sequences were acquired to detect brain abnormalities.

**Fig. 1:**
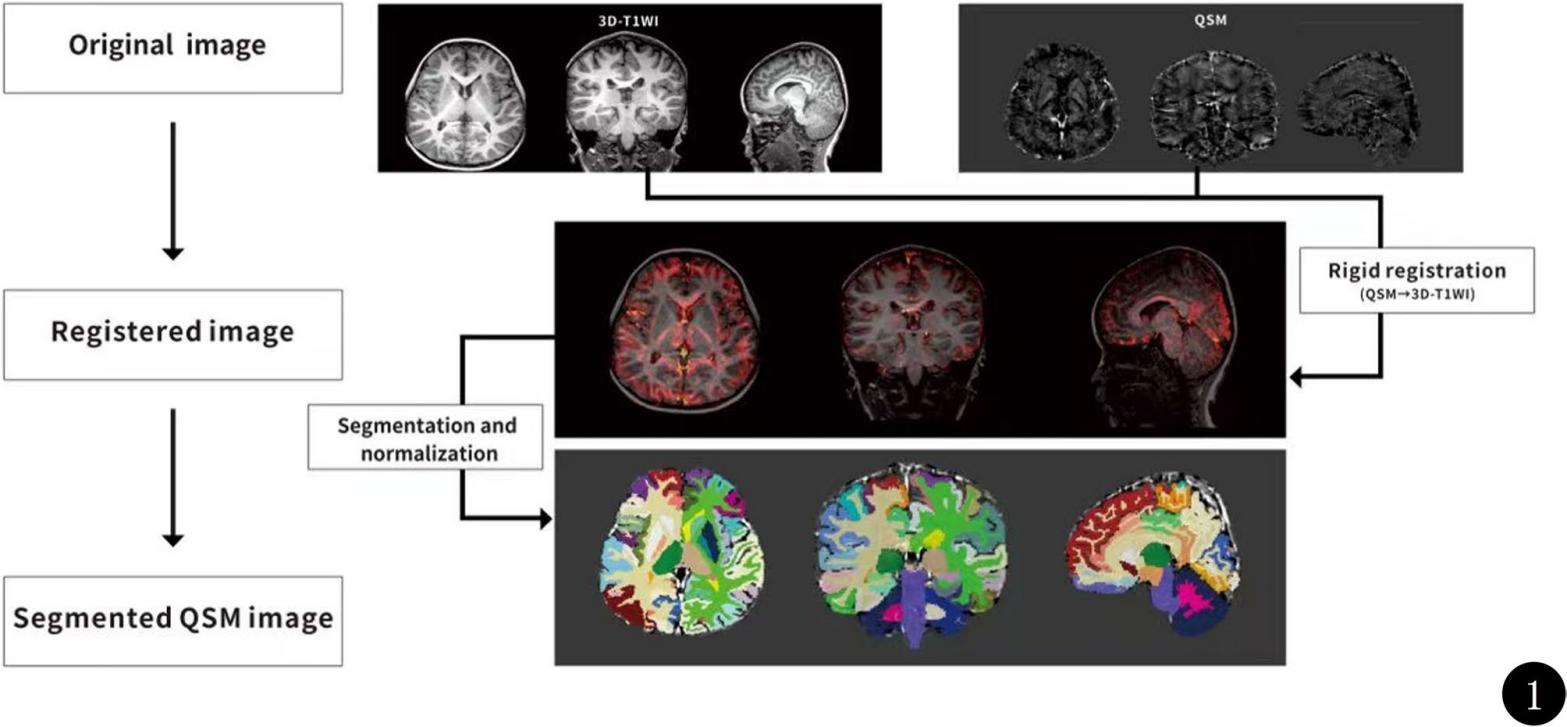
Schematic diagram of brain segmentation and measurement of QSM data. QSM, quantitative susceptibility mapping

The STI Suite toolbox version 3.0 (https://people.eecs.berkeley.edu/~chunlei.liu/software.html) on the MATLAB R2018b (Mathworks) platform was used to calculate the susceptibility maps (QSM images) from the phase images through the following steps: phase unwrapping, background field removal, and tissue susceptibility calculation. Specific processing steps are detailed in previous research [24, 25]. Brain segmentation and QSM value extraction were performed using an in-house developed tool, BrainQuanAll (Chengdu Zhongying Medical Technology Co., Ltd.), as shown in Fig. 1. BrainQuanAll, which is based on Python 3.8 and FreeSurfer, has been applied in previous studies [26, 27]. The software followed the parameters recommended by FreeSurfer, using both the cross-sectional and longitudinal streams. First, the QSM images were rigidly registered to the 3D-T1WI images. Then, the 3D-T1WI images were segmented and non-linearly normalized into the MNI space to produce the tissue probability maps and normalized QSM quantitative maps. Finally, the brain was automatically segmented and the magnetic susceptibility value of each brain structure was extracted.

### Statistical analysis

The statistical analysis was performed utilizing SPSS Statistics version 25.0 software (IBM Corp.). Initially, all quantitative data were examined for normality using the Shapiro-Wilk test. Quantitative data with normal distribution were expressed as mean ± standard deviation and compared by two independent samples t test. Data that were not normally distributed were expressed as median (interquartile range) and compared using the Mann-Whitney U test. Categorical variables were analyzed using the Chi-Squared test. Partial correlation analysis was utilized to evaluate the relationships between the GDS scores and the susceptibility values in each brain region that showed significant differences in the ASD group after controlling for age and sex effects. Additionally, the diagnostic ability of susceptibility values in differentiating ASD patients and TD children was evaluated using the receiver operating characteristic (ROC) curves and the area under the curve (AUC). A significance threshold of *p* < 0.05 was employed for all analyses. FDR correction was used to correct for multiple comparisons.

## Results

### Demographic data

**Fig. 2:**
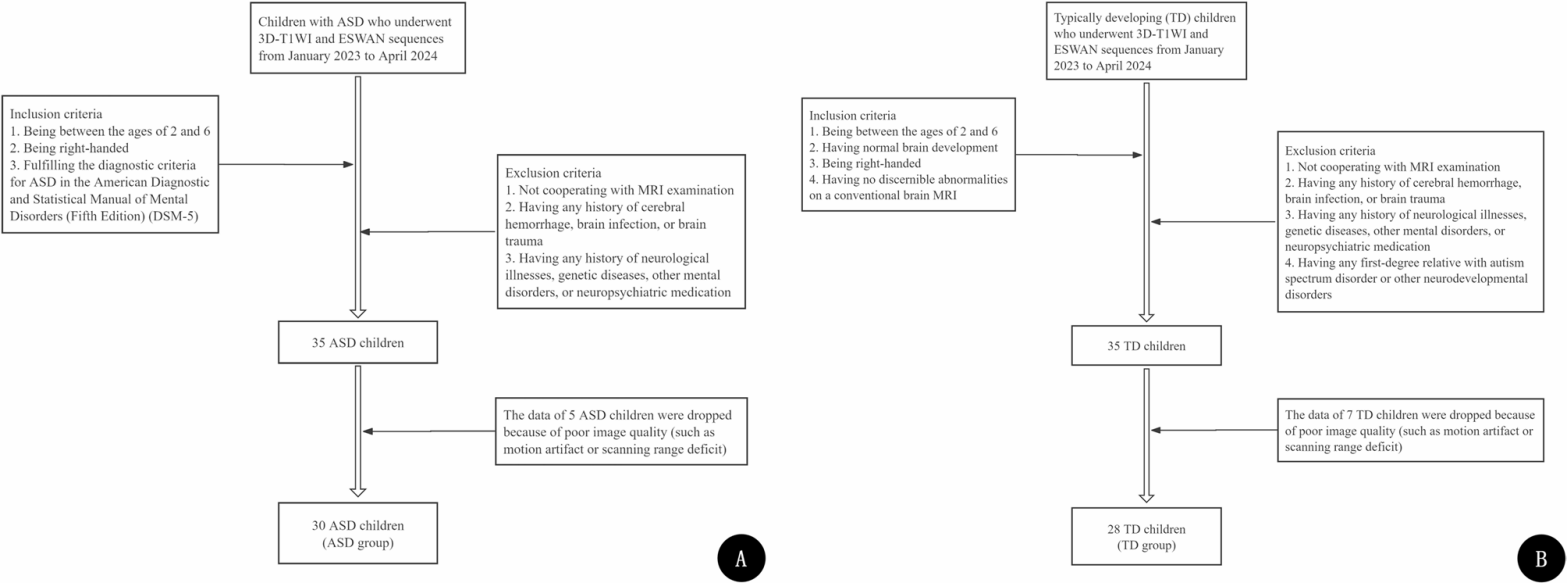
Flowchart of participant inclusion and exclusion. **A** participant inclusion and exclusion criteria for ASD patients; **B** participant inclusion and exclusion criteria for TD children. ASD, autism spectrum disorder; TD, typically developing

After 5 ASD patients and 7 TD children were excluded because of poor image quality (such as motion artifact or scanning range deficit), 30 ASD and 28 TD participants were included in this study (Fig. 2). Characteristics of the study population are presented in Table 1, with no significant differences in sex ratio or age between groups (*p* > 0.05).

**Table 1:** Demographic data of all ASD and TD children

	ASD group(*n* = 30)	TD group(*n* = 28)	t/x^2^ value	*p* value
Sex(male/female)	22/8	14/14	3.35	0.07^a^
Age(months)	48.70 ± 11.76	44.93 ± 9.19	0.06	0.18^b^

*ASD* autism spectrum disorder, *TD* typically developing

^a^Statistical analysis was performed using x^2^ test

^b^Two-independent-samples t test was used for statistical analysis

*p* < 0.05 was defined as the significance level

### The result of QSM analysis

**Fig. 3:**
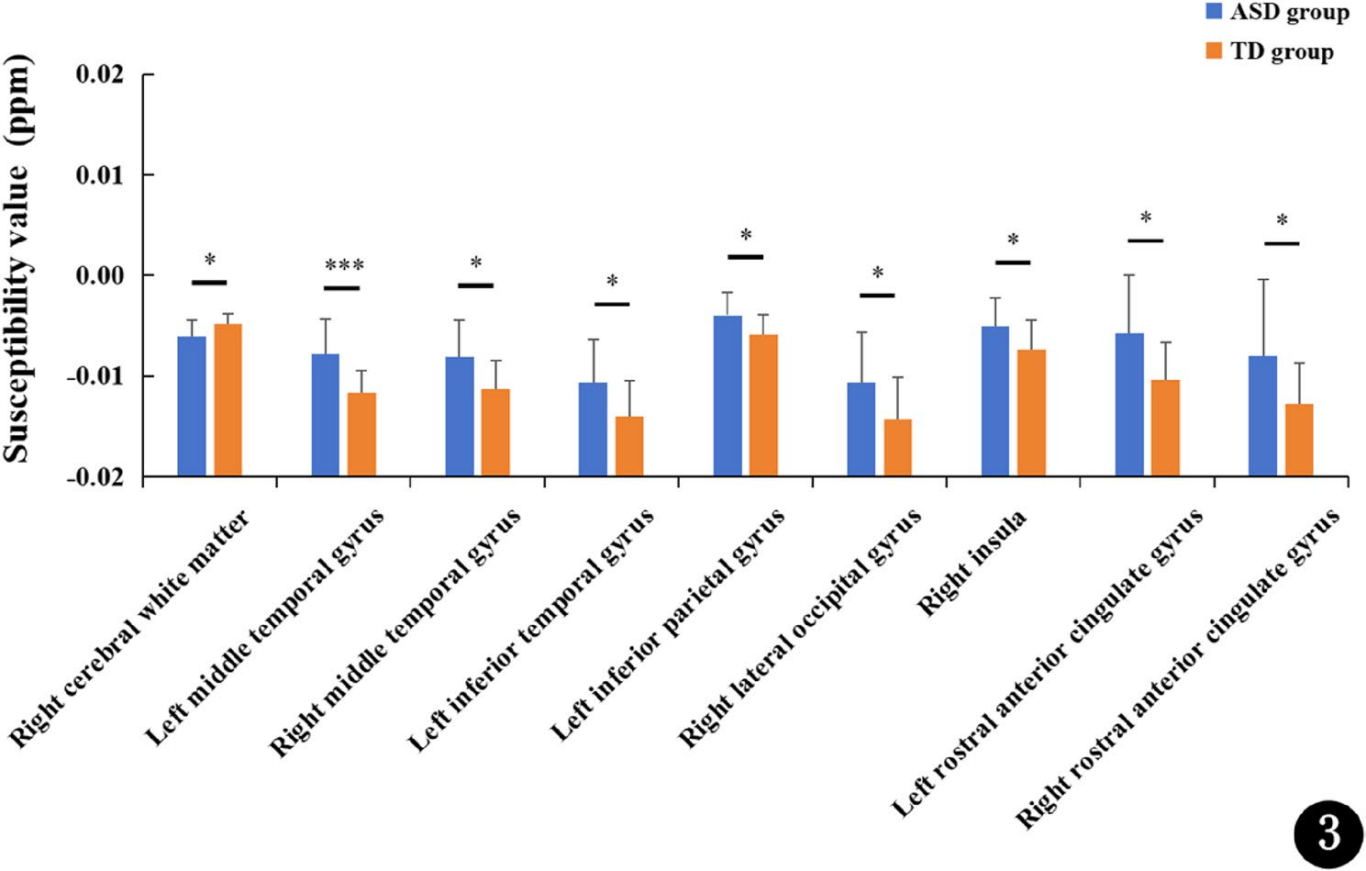
Comparisons of susceptibility values between ASD and TD children. ASD, autism spectrum disorder; TD, typically developing; QSM, quantitative susceptibility mapping; *:*p* < 0.05; ***:*p* < 0.001

Fig. 3 shows the comparison of susceptibility values between ASD and TD children. Compared to TD children, the ASD children exhibited significantly increased susceptibility in the bilateral middle temporal gyrus, left inferior temporal gyrus, left inferior parietal gyrus, right lateral occipital gyrus, right insula, and bilateral rostral anterior cingulate gyrus (*p* < 0.05, FDR correction). Conversely, significantly reduced susceptibility was observed in the right cerebral white matter (*p* < 0.05, FDR correction).

### Correlation analysis

In the correlation analysis, susceptibility values of the left middle temporal gyrus, left inferior parietal gyrus, and right lateral occipital gyrus showed significant negative correlations with GDS-gross motor DQ in the ASD group (*r* = −0.384, *p* = 0.044; *r* = −0.427, *p* = 0.023; *r* = −0.377, *p* = 0.048) (Table 2 and Supplementary material, Fig. 1). The correlations, though not significant after FDR correction, suggest a trend associating susceptibility values with neurodevelopmental state in ASD. No significant correlations were found between susceptibility values in any ROI and DQ scores in the other four GDS domains: adaptive behavior, fine motor, language, or personal-social behavior. The corresponding results are presented in the Supplementary material (Tables S1-S5). The statistical threshold was set at uncorrected *p* < 0.05 due to the exploratory nature of these analyses.

**Table 2:** Correlations between susceptibility values and GDS-gross motor scale scores in ASD children (partial correlation, age and sex controlled)

Brain regions	*r* value	*p* value
Right cerebral white matter	0.317	0.100
Left middle temporal gyrus	−0.384	0.044^*^
Right middle temporal gyrus	−0.146	0.458
Left inferior temporal gyrus	−0.208	0.287
Left inferior parietal gyrus	−0.427	0.023^*^
Right lateral occipital gyrus	−0.377	0.048^*^
Right insula	−0.267	0.170
Left rostral anterior cingulate gyrus	−0.139	0.480
Right rostral anterior cingulate gyrus	−0.141	0.473

*GDS* Gesell developmental schedules, *ASD* autism spectrum disorder

*uncorrected *p* < 0.05

### ROC curve analysis

**Fig. 4:**
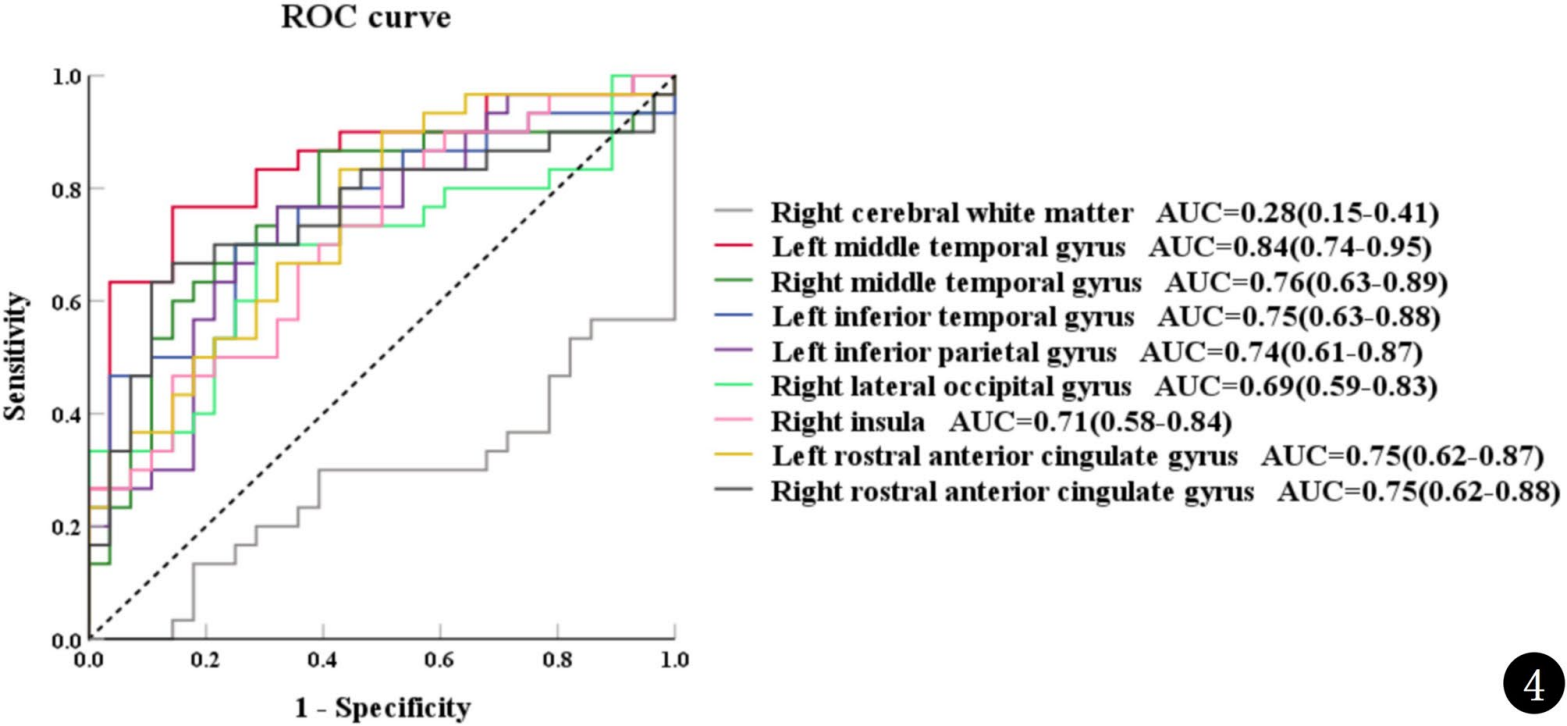
The ROC curve was generated using the susceptibility values of brain regions with significant differences for distinguishing ASD patients from TD children. The susceptibility value of the left middle temporal gyrus had a larger area under the curve (AUC = 0.84). ROC, receiver operating characteristic; AUC, area under the curve; ASD, autism spectrum disorder; TD, typically developing

ROC curves were generated using the bilateral middle temporal gyrus, left inferior temporal gyrus, left inferior parietal gyrus, right lateral occipital gyrus, right insula, bilateral rostral anterior cingulate gyrus, and right cerebral white matter to assess their potential as biomarkers for differentiating between TD children and ASD children (Fig. 4). The AUCs of these nine brain regions were 0.84, 0.76, 0.75, 0.74, 0.69, 0.71, 0.75, 0.75, and 0.28, respectively.

## Discussion

ESWAN, a new technique built upon SWI, combines three-dimensional T2*-based multi-echo acquisition with a reconstruction algorithm [28]. Because of its high sensitivity to magnetic susceptibility, ESWAN provides high spatial resolution and signal-to-noise ratio for tissue imaging. Iron deposition appears as localized low-intensity regions on magnitude images, while phase images reveal tissue susceptibility changes due to iron. Moreover, phase values reflect iron deposition more accurately than magnitude values due to their insensitivity to relaxation time or radiofrequency field variations [29]. The QSM technique quantitatively computes magnetic susceptibility values from preprocessed phase data by analyzing minute changes in magnetic field strength through field-to-source inversion [23, 30]. In the brain, the susceptibility contrast has been demonstrated to originate primarily from two sources: iron and myelin [31, 32]. Although both iron and myelin theoretically affect susceptibility values in opposite directions, the susceptibility values of white matter are consistent with the known characteristics of brain myelination during brain maturation, and the exponential growth of susceptibility contrast in gray matter is also in good agreement with the known characteristics of iron deposition [21]. Therefore, we can reasonably infer that the primary cause of elevated susceptibility in gray matter in our results is increased iron content, while the primary cause of reduced susceptibility in white matter is increased myelination.

Through whole-brain susceptibility comparisons between ASD and TD children, we discovered widespread abnormal iron content in cortical regions. Specifically, we found significantly higher mean susceptibility values in ASD patients compared to TD children in the bilateral middle temporal gyrus, left inferior temporal gyrus, left inferior parietal gyrus, right lateral occipital gyrus, right insula, and bilateral rostral anterior cingulate gyrus. ASD children showed significantly decreased susceptibility values in the right cerebral white matter, which was roughly consistent with the results of earlier research in which the ROIs were manually delineated [22, 23]. Second, we discovered potential negative correlations between clinical features and elevated QSM values in certain brain regions. Furthermore, the susceptibility values in some of these areas exhibited good capacity to differentiate ASD patients from TD individuals. These findings provided insights and direction for future studies in ASD patients from the perspective of whole-brain iron content.

The mechanisms responsible for elevated cerebral iron levels remain incompletely understood. One proposed explanation involves disrupted brain iron homeostasis in children with ASD; specifically, increased iron accumulation may result from dysregulation of iron-regulatory proteins [8]. Previous QSM studies have primarily reported reduced brain iron levels in pediatric developmental disorders and proposed explanations for how iron deficiency contributes to pathophysiology [9, 14]. However, understanding of the pathophysiological role of elevated brain tissue iron in developing populations remains limited. A Mendelian randomization study suggested that increased brain iron in ASD patients may contribute to ASD risk through inevitable triggering of oxidative stress and ferroptosis [33]. Multiple studies have found links between ASD and elevated oxidative stress, while recent studies reported aberrant expression of ferroptosis-related genes in ASD samples compared to controls [34–36]. Excess iron generates substantial reactive oxygen species, increasing oxidative damage and inducing ferroptosis-pathological processes that may underlie core clinical manifestations of ASD [37–41]. However, these mechanistic interpretations require further experimental validation.

In the current study, ASD patients showed elevated susceptibility values in bilateral middle temporal gyri, which were involved in visual motion and egomotion processing in primates and humans [42–45]. A functional magnetic resonance imaging (fMRI) analysis revealed that the magnitude of middle temporal area activation was associated with autism severity [46]. Consistent with these results, we discovered a potential negative correlation between left middle temporal gyrus susceptibility values and GDS-gross motor scores, suggesting that excessive iron deposition in the left middle temporal gyrus would be related to abnormal motor regulation in ASD. Additionally, the ROC curve revealed that the left middle temporal gyrus susceptibility value had a larger AUC. This suggested that the left middle temporal gyrus susceptibility values demonstrated a high capacity to distinguish between ASD patients and TD children. Consequently, additional research is required to explore this issue in depth.

Another significant discovery from our investigation was abnormal iron accumulation in the left inferior parietal gyrus. It has been repeatedly demonstrated that the inferior parietal lobule played a crucial role in motor skill representation [47–49], dexterous manipulation for tool-related and communicative gestures [47, 50], and high-level action processing including motor imagery and action comprehension [51–53]. Structural alterations in this area correlated with poor motor performance in ASD children [54], while functional connectivity abnormalities were linked to impaired praxis and social-communicative abilities [55]. Similarly, we observed increased susceptibility values in the inferior parietal gyrus of ASD patients, potentially correlating with lower GDS-gross motor DQ scores. This suggested that aberrant iron accumulation may contribute to motor dysfunction. However, the specific pathogenesis still needs further study.

The lateral occipital gyrus contributes to higher-order functions including object recognition, facial recognition, and motion perception [56]. ASD patients exhibited distinct visual perception patterns compared to healthy controls: they showed superior performance in detecting static visual targets within complex backgrounds, but demonstrated reduced sensitivity to complex motion stimuli [57–61]. In addition, a recent study showed that reduced resting-state activity and structural connectivity in this region correlated with social communication deficits in ASD boys [62]. A meta-analysis of VBM studies also indicated structural anomalies of the lateral occipital gyrus in ASD individuals [57]. In the present study, we detected increased susceptibility values in the right lateral occipital gyrus of ASD patients, with an inverse correlation to GDS-gross motor scores. This further confirmed the significance of the lateral occipital gyrus in the pathogenesis of ASD. Hence, it would be worthwhile to further investigate the underlying mechanism in subsequent research.

Several limitations should be noted in this work. First, this was a small-scale, single-center study, so we will increase the sample size and conduct a multi-center study in the future to strengthen the statistical power and verify the results of this study. Second, this cross-sectional investigation was unable to reveal dynamic variations in iron levels of ASD children; thus, longitudinal follow-up is required to provide valuable insights into the evolution of QSM biomarkers over time in ASD. Third, although iron dominates magnetic susceptibility in brain tissue, other metal elements (e.g., calcium, copper) may subtly influence measurements. Current QSM methodology cannot disentangle iron from these confounding factors-a fundamental technical limitation. Moreover, the high myelin content in white matter inherently obscures accurate iron quantification, necessitating advanced techniques like DECOMPOSE-QSM, χ-separation imaging, and APART-QSM for future research [32, 63, 64]. Fourth, ethical restrictions precluded recording dietary iron intake or serum ferritin levels, factors that may influence brain iron measurements and warrant consideration in future studies. Finally, while identical sedation protocols minimized inter-group bias, residual physiological effects of sedation cannot be excluded. Thus, unsedated QSM acquisition is recommended where clinically feasible.

## Conclusions

In conclusion, our study revealed that ASD children had aberrant susceptibility values in cortical areas. The susceptibility values of some brain regions were related to the severity of neurodevelopmental disorder. Additionally, the susceptibility value of the left middle temporal gyrus may be a potential imaging biomarker for the early detection of ASD. These findings provided new insights into the understanding of ASD, particularly in terms of neurobiology, and might suggest new directions for future studies.

## Supplementary Information

Supplementary Material 1.

Supplementary Material 2.

Supplementary Material 3.

Supplementary Material 4.

## Data Availability

The datasets generated and/or analyzed during the current study are not publicly available due to privacy and ethical restrictions but are available from the corresponding author on reasonable request.

## Abbreviations

ROIs: Regions of interest
ASD: Autism spectrum disorder
QSM: Quantitative susceptibility mapping
TD: Typically developing
GDS: Gesell developmental schedules
ESWAN: Enhanced susceptibility-weighted angiography
DSM-5: The Diagnostic and Statistical Manual of Mental Disorders (Fifth Edition)
DQ: Developmental quotient
TR: Repetition time
TE: Echo time
FA: Flip angle
FOV: Field of view
ROC curve: Receiver operating characteristic curve
AUC: Area under curve
