# Supplementary material for: Quantitative susceptibility mapping shows alterations of brain iron content in children with autism spectrum disorder: a whole-brain analysis
Source: BMC Psychiatry. 2025 Aug 27;25:826. doi: 10.1186/s12888-025-07235-y (PMC12392503; doi:10.1186/s12888-025-07235-y)
Supplement: Supplementary file 1 — Supplementary Material 1. [file 12888_2025_7235_MOESM1_ESM.docx]

**
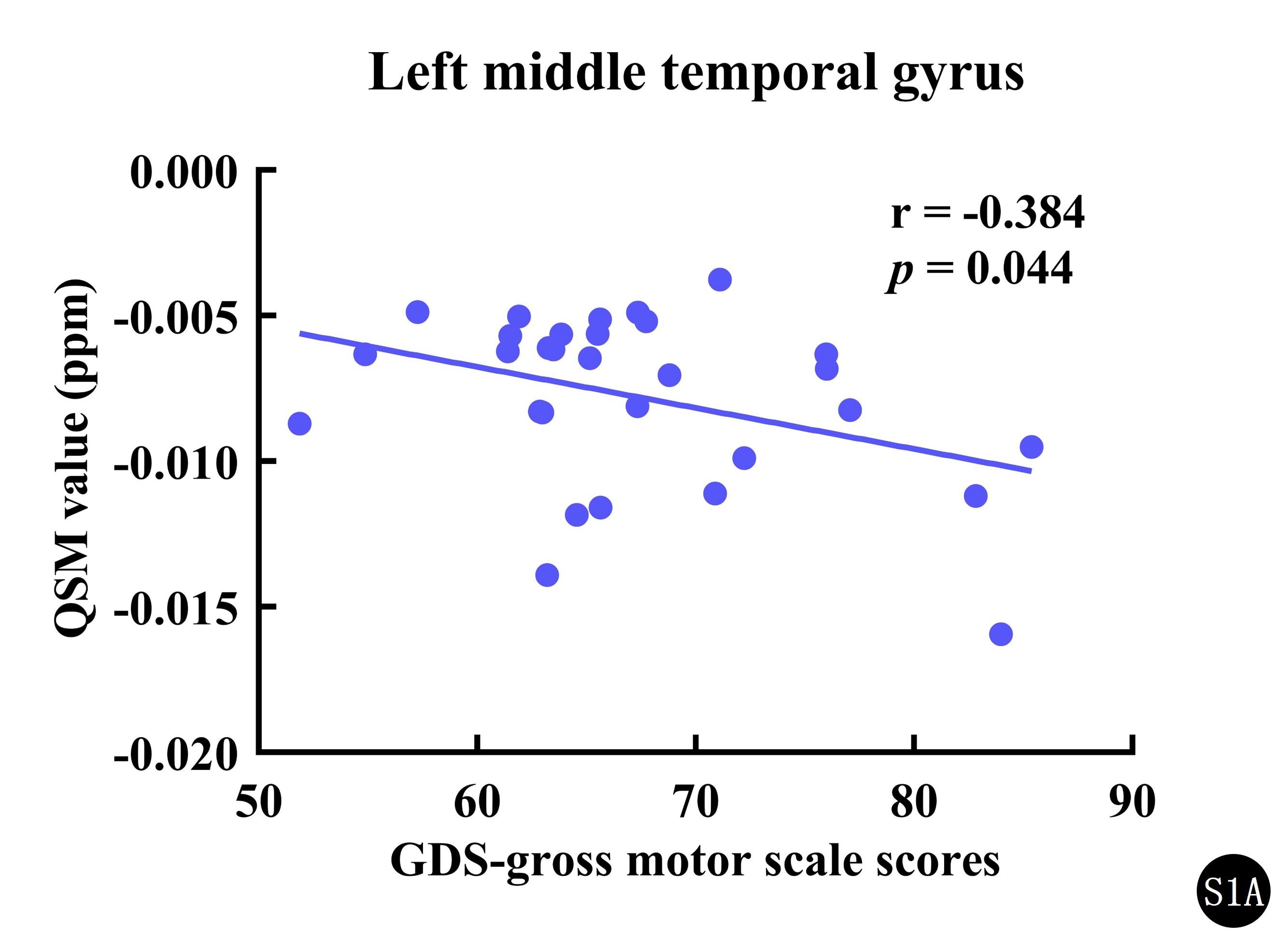
**
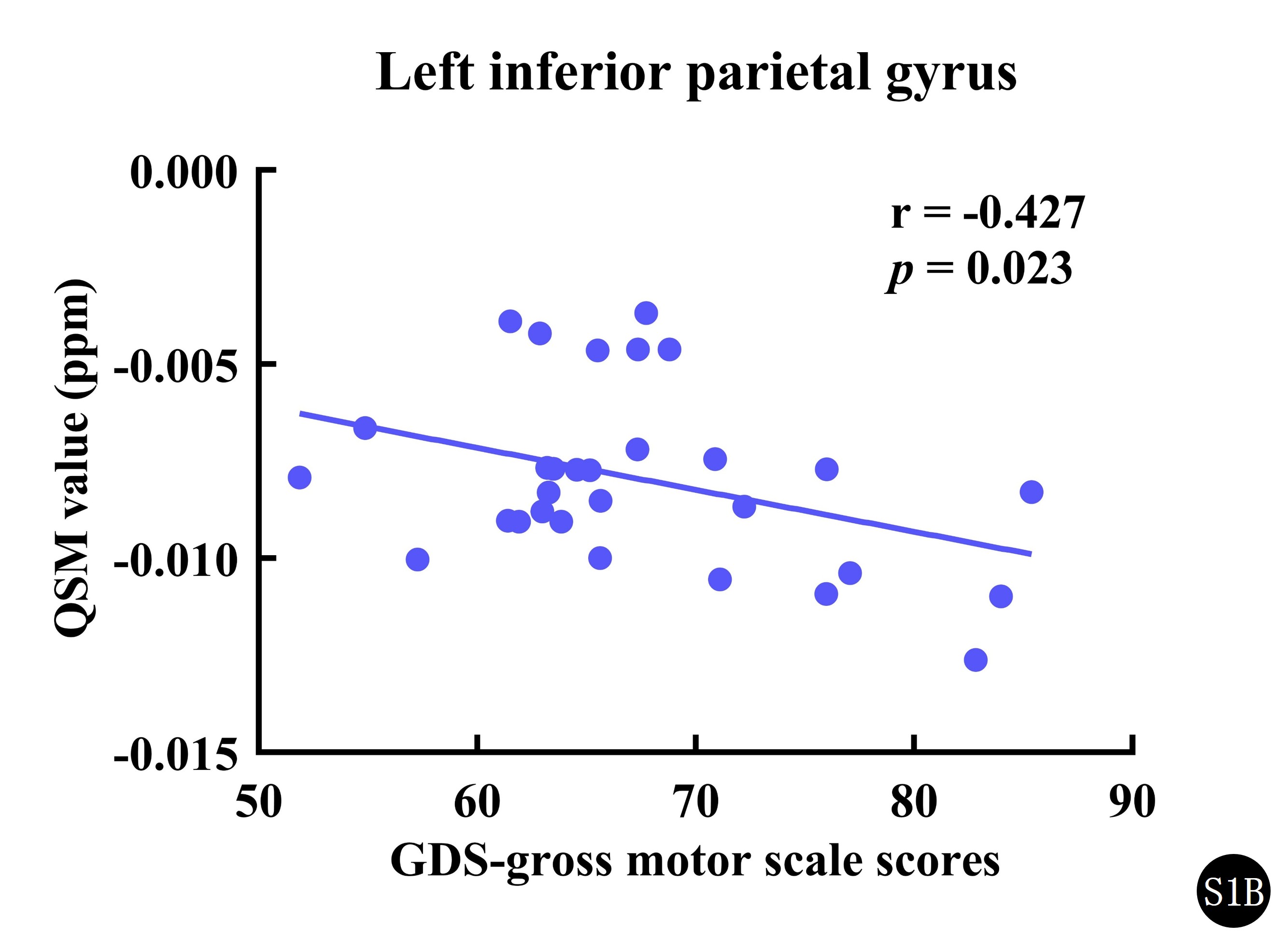


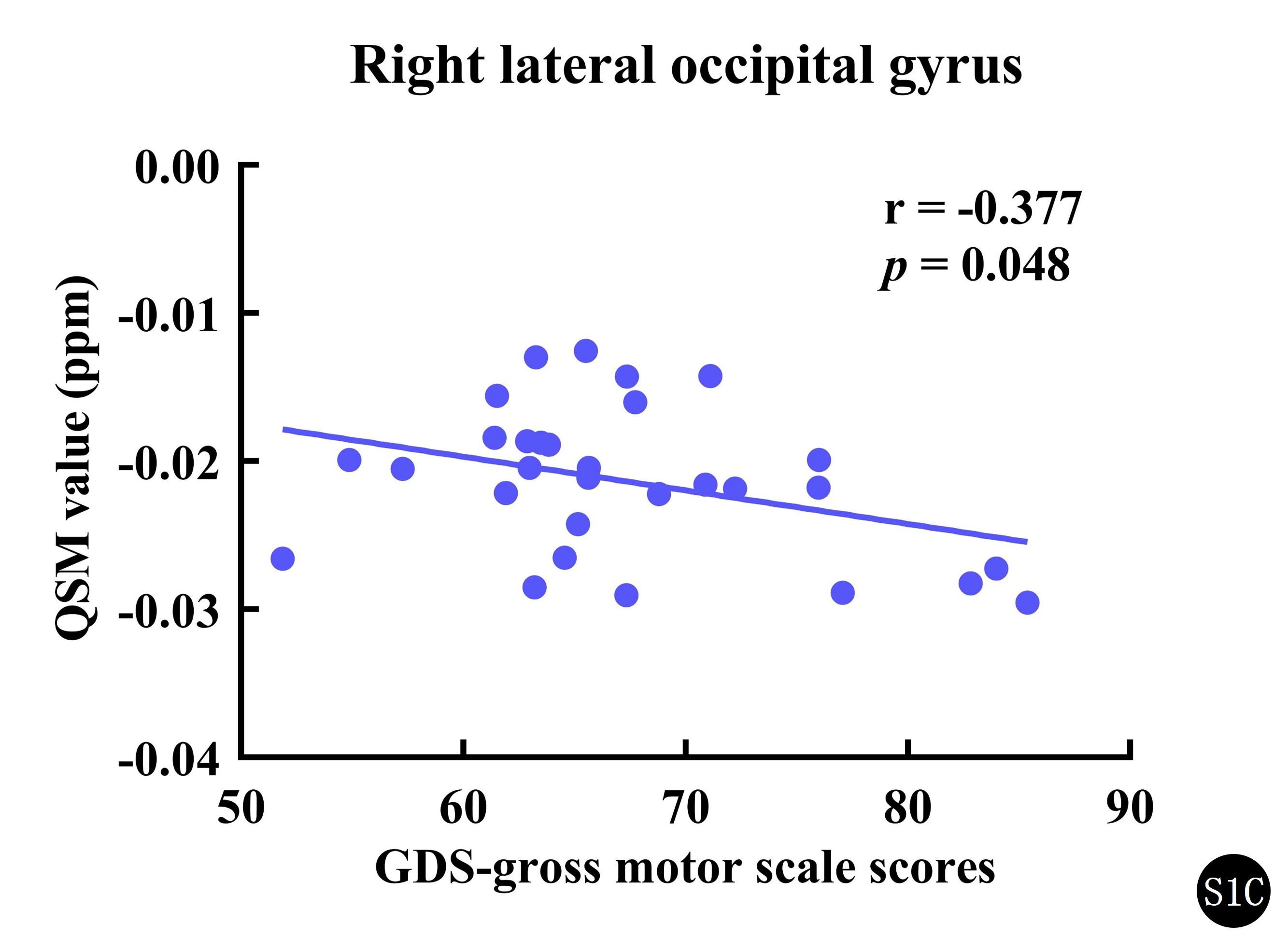


**Supplementary Figure 1.** Correlation analyses in ASD subjects (partial correlation, age and sex controlled). S1A: the correlation between the QSM values of the left middle temporal gyrus and the GDS-gross motor scale scores; S1B: the correlation between the QSM values of the left inferior parietal gyrus and the GDS-gross motor scale scores; S1C: the correlation between the QSM values of the right lateral occipital gyrus and the GDS-gross motor scale scores. ASD, autism spectrum disorder; QSM, quantitative susceptibility mapping; GDS, Gesell developmental schedules; uncorrected *p* < 0.05.

**Supplementary Table 1.** Correlations between susceptibility values and GDS- adaptive behavior scale scores in ASD children (partial correlation, age and sex controlled).

| Brain regions | r value | 95% CI (r) | *p* value |
| --- | --- | --- | --- |
| Right cerebral white matter | 0.084 | (-0.299, 0.443) | 0.670 |
| Left middle temporal gyrus | -0.084 | (-0.443, 0.299) | 0.671 |
| Right middle temporal gyrus | -0.040 | (-0.407, 0.338) | 0.840 |
| Left inferior temporal gyrus | -0.139 | (-0.486, 0.247) | 0.480 |
| Left inferior parietal gyrus | 0.055 | (-0.325, 0.419) | 0.780 |
| Right lateral occipital gyrus | -0.142 | (-0.488, 0.244) | 0.472 |
| Right insula | -0.143 | (-0.489, 0.243) | 0.468 |
| Left rostral anterior cingulate gyrus | -0.203 | (-0.537, 0.184) | 0.301 |
| Right rostral anterior cingulate gyrus | -0.074 | (-0.435, 0.308) | 0.708 |
| GDS, Gesell developmental schedules; ASD, autism spectrum disorder; *: uncorrected *p* < 0.05. | | | |

**Supplementary Table 2.** Correlations between susceptibility values and GDS-gross motor scale scores in ASD children (partial correlation, age and sex controlled).

| Brain regions | r value | 95% CI (r) | *p* value |
| --- | --- | --- | --- |
| Right cerebral white matter | 0.317 | (-0.064, 0.617) | 0.100 |
| Left middle temporal gyrus | -0.384 | (-0.663, -0.012) | 0.044^*^ |
| Right middle temporal gyrus | -0.146 | (-0.491, 0.240) | 0.458 |
| Left inferior temporal gyrus | -0.208 | (-0.540, 0.179) | 0.287 |
| Left inferior parietal gyrus | -0.427 | (-0.690, -0.064) | 0.023^*^ |
| Right lateral occipital gyrus | -0.377 | (-0.659, -0.005) | 0.048^*^ |
| Right insula | -0.267 | (-0.579, 0.118) | 0.170 |
| Left rostral anterior cingulate gyrus | -0.139 | (-0.486, 0.247) | 0.480 |
| Right rostral anterior cingulate gyrus | -0.141 | (-0.488, 0.245) | 0.473 |
| GDS, Gesell developmental schedules; ASD, autism spectrum disorder; *: uncorrected *p* < 0.05. | | | |

**Supplementary Table 3.** Correlations between susceptibility values and GDS- fine motor scale scores in ASD children (partial correlation, age and sex controlled).

| Brain regions | r value | 95% CI (r) | *p* value |
| --- | --- | --- | --- |
| Right cerebral white matter | -0.016 | (-0.387, 0.359) | 0.937 |
| Left middle temporal gyrus | 0.071 | (-0.311, 0.433) | 0.721 |
| Right middle temporal gyrus | 0.042 | (-0.337, 0.409) | 0.833 |
| Left inferior temporal gyrus | -0.135 | (-0.483, 0.250) | 0.494 |
| Left inferior parietal gyrus | 0.213 | (-0.173, 0.543) | 0.276 |
| Right lateral occipital gyrus | 0.109 | (-0.274, 0.464) | 0.581 |
| Right insula | 0.116 | (-0.268, 0.469) | 0.556 |
| Left rostral anterior cingulate gyrus | 0.051 | (-0.328, 0.416) | 0.795 |
| Right rostral anterior cingulate gyrus | 0.025 | (-0.351, 0.395) | 0.898 |
| GDS, Gesell developmental schedules; ASD, autism spectrum disorder; *: uncorrected *p* < 0.05. | | | |

**Supplementary Table 4.** Correlations between susceptibility values and GDS- language scale scores in ASD children (partial correlation, age and sex controlled).

| Brain regions | r value | 95% CI (r) | *p* value |
| --- | --- | --- | --- |
| Right cerebral white matter | -0.178 | (-0.518, 0.209) | 0.366 |
| Left middle temporal gyrus | -0.016 | (-0.387, 0.359) | 0.935 |
| Right middle temporal gyrus | 0.025 | (-0.351, 0.395) | 0.901 |
| Left inferior temporal gyrus | 0.037 | (-0.342, 0.405) | 0.851 |
| Left inferior parietal gyrus | -0.105 | (-0.459, 0.280) | 0.594 |
| Right lateral occipital gyrus | -0.107 | (-0.462, 0.277) | 0.587 |
| Right insula | -0.275 | (-0.590, 0.109) | 0.157 |
| Left rostral anterior cingulate gyrus | -0.131 | (-0.480, 0.254) | 0.506 |
| Right rostral anterior cingulate gyrus | 0.045 | (-0.334, 0.411) | 0.819 |
| GDS, Gesell developmental schedules; ASD, autism spectrum disorder; *: uncorrected *p* < 0.05. | | | |

**Supplementary Table 5.** Correlations between susceptibility values and GDS- personal-social behavior scale scores in ASD children (partial correlation, age and sex controlled).

| Brain regions | r value | 95% CI (r) | *p* value |
| --- | --- | --- | --- |
| Right cerebral white matter | -0.211 | (-0.543, 0.176) | 0.281 |
| Left middle temporal gyrus | 0.214 | (-0.173, 0.543) | 0.273 |
| Right middle temporal gyrus | 0.064 | (-0.317, 0.427) | 0.746 |
| Left inferior temporal gyrus | 0.253 | (-0.132, 0.571) | 0.193 |
| Left inferior parietal gyrus | 0.185 | (-0.202, 0.521) | 0.346 |
| Right lateral occipital gyrus | 0.251 | (-0.134, 0.569) | 0.198 |
| Right insula | 0.031 | (-0.347, 0.400) | 0.877 |
| Left rostral anterior cingulate gyrus | 0.156 | (-0.231, 0.500) | 0.428 |
| Right rostral anterior cingulate gyrus | 0.157 | (-0.230, 0.501) | 0.426 |
| GDS, Gesell developmental schedules; ASD, autism spectrum disorder; *: uncorrected *p* < 0.05. | | | |
