# Supplementary figures and images for: Quantitative susceptibility mapping shows alterations of brain iron content in children with autism spectrum disorder: a whole-brain analysis
Source: BMC Psychiatry. 2025 Aug 27;25:826. doi: 10.1186/s12888-025-07235-y (PMC12392503; doi:10.1186/s12888-025-07235-y)

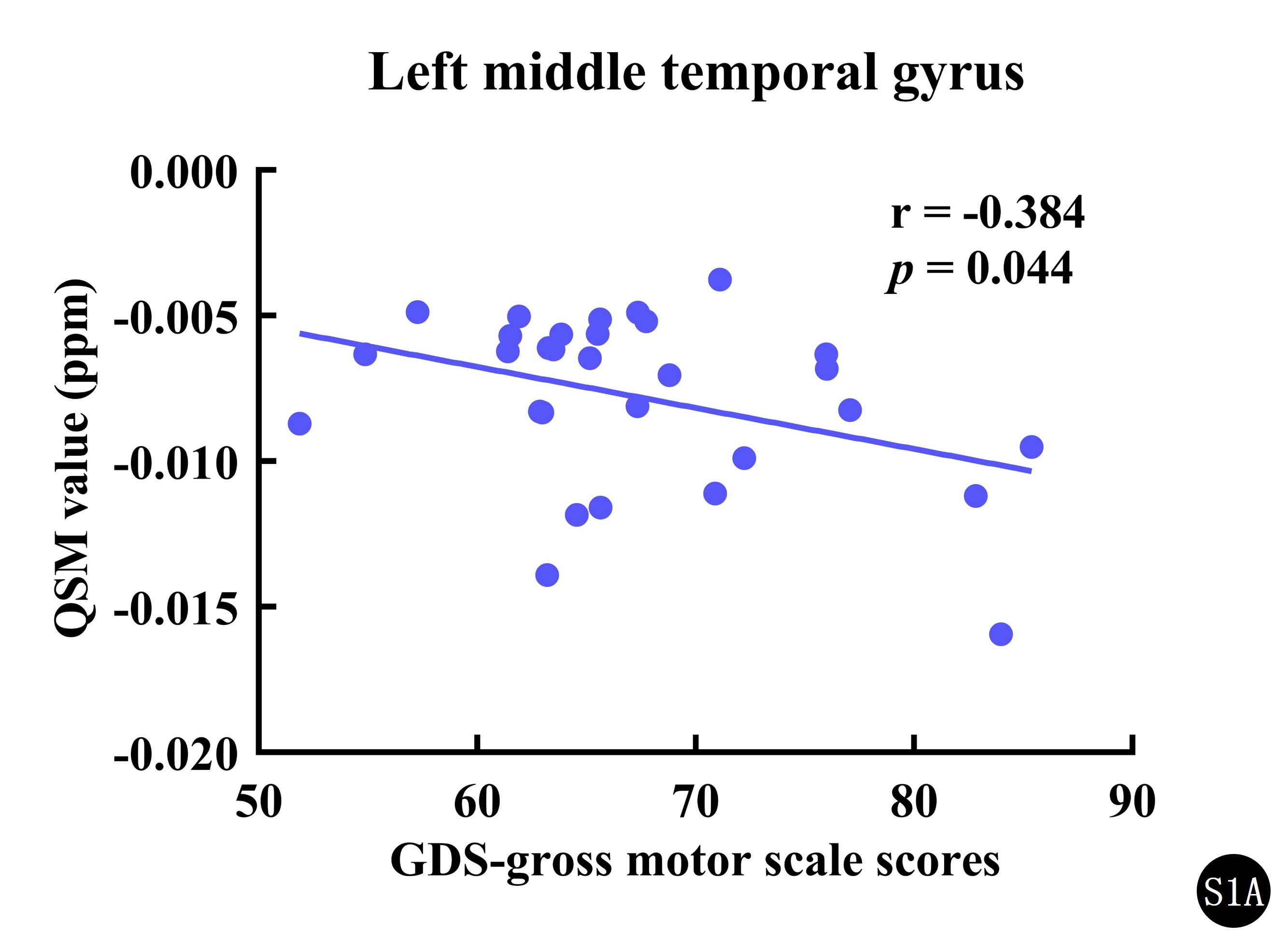

Supplement: Supplementary file 2 — Supplementary Material 2. [file 12888_2025_7235_MOESM2_ESM.jpg]

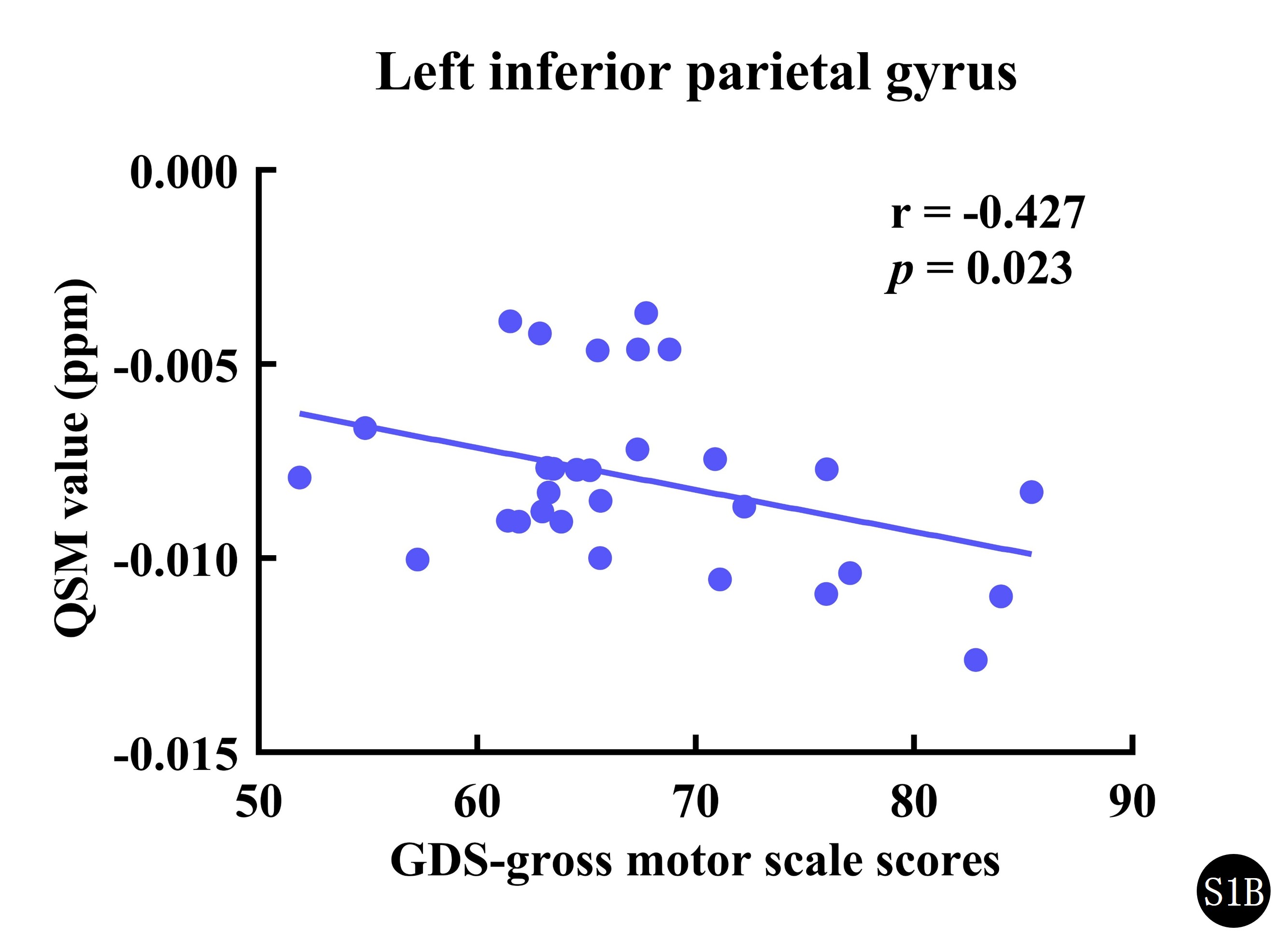

Supplement: Supplementary file 3 — Supplementary Material 3. [file 12888_2025_7235_MOESM3_ESM.jpg]

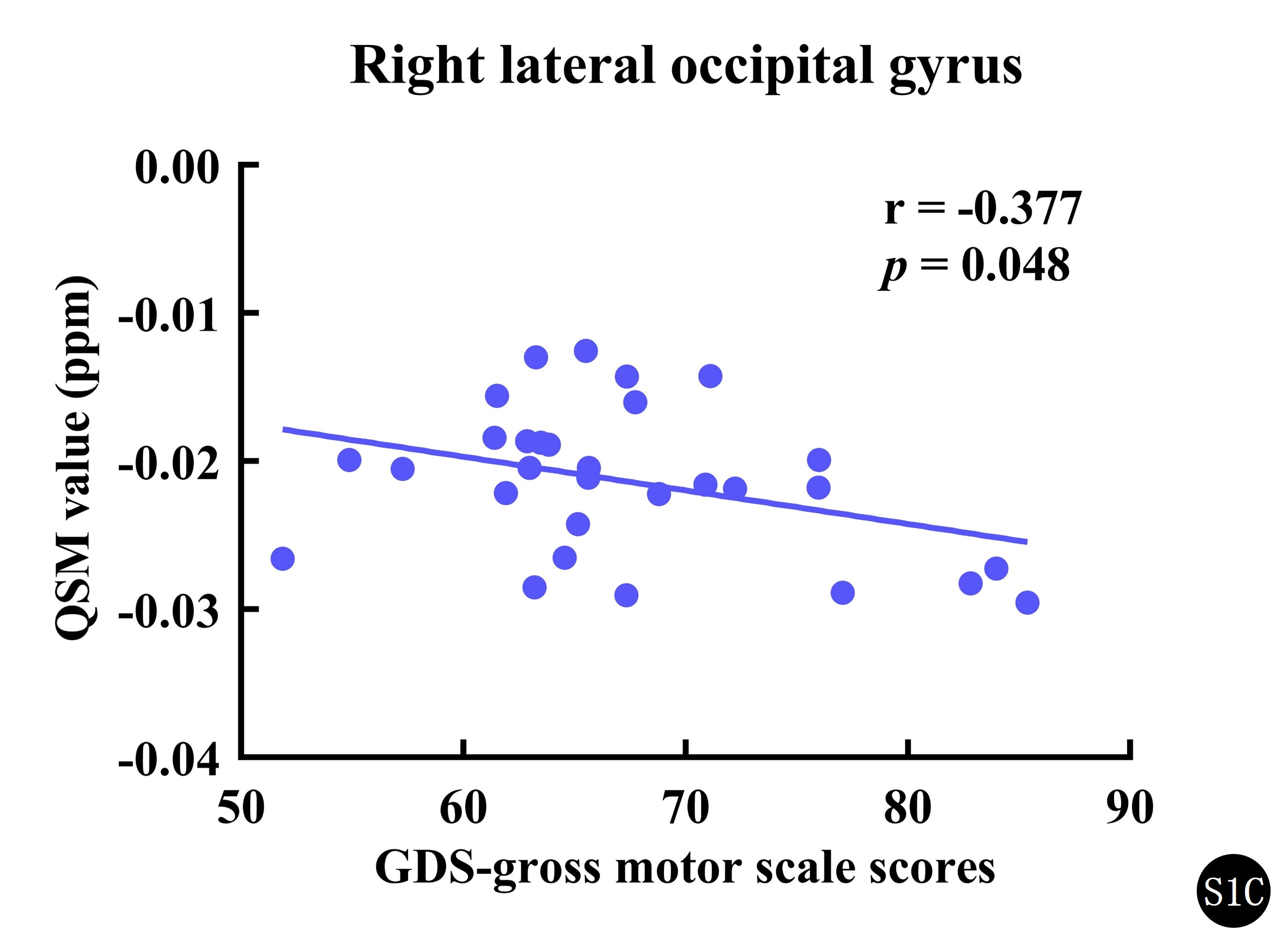

Supplement: Supplementary file 4 — Supplementary Material 4. [file 12888_2025_7235_MOESM4_ESM.jpg]
